# The Treatment of Glottic Spasm

**Published:** 1909-06-26

**Authors:** 


### LARYNGOLOGY AND RHINOLOGY.

### THE TREATMENT OF GLOTTIC SPASM.

THE various forms of spasm of the glottis were recently discused in THE HOSPITAL (June 5, 1909, p. 263). Their treatment naturally comes under two headings, relief of the actual spasm and prevention of the attacks. In spasm of the glottis in adults, the attack is generally short and sharp, and leaves little time for direct treatment; the inhalation of nitrite of amyl frequently gives relief, and the patient subject to these attacks should be directed to carry a 5-minim capsule of this drug for immediate use if required. When the attacks are less acute but more persistent, the administration of an emetic dose of ipecacuanha is the most reliable method of allaying the spasm; 30 grains of the powdered root, or four to six drachms of the vinum ipecacuanhæ should be given. Except when due to serious disease, as locomotor ataxia or thoracic aneurysm, these attacks of spasm are seldom dangerous, though they are very alarming to the patient; tracheotomy or intubation is practically never required. The spasm always disappears under chloroform, which may be administered in bad cases by the medical attendant; it need not be given to the extent of inducing anæsthesia. In the persistent cases, also, a dose of chloral may give relief.

For the preventive treatment the exciting cause must be found and, when possible, treated. All sources of irritation, such as smoking, must be avoided. In some patients a spasm is readily induced by drinking cold fluids, and this must then, of course, be forbidden. A general tonic and hygienic *régime* is indicated, and change of air and scene is often of great value. Little can be hoped for from drugs, but a course of treatment by bromides sometimes has a good effect.

The quickest and surest method of relieving the spasm of *Laryngismus Stridulus* is to hook the epiglottis forward with the finger, and the mother or nurse can easily be taught to do this. The spasm also often ceases on stimulating the conjunctiva by touching it with the finger, or the nasal mucous membrane by tickling it with a feather. Smelling salts or ammonia held under the nose may have the same effect, or the inhalation of 1 to 3 minims of amyl nitrite may be tried. Pouring cold water on to the head while the child is placed in a hot bath has been recommended, but there is little time to do this, as the attacks are very short and acute. It should be remembered that they may prove fatal, although this is decidedly uncommon. The preventive treatment consists in the general treatment of rickets if present, and attention to the diet and general health. Overloading of the stomach must be carefully avoided, the food must be good in quality, moderate in quantity, and given at regular intervals, and the regular action of the bowels must be ensured. A change of air is often most useful, especially to a higher altitude. Moritz Schmidt relates a case in which he found immediate benefit by moving the child to the top of the house.

In the *Spasmodic Laryngitis* of childhood, the spasm should be treated on the same lines as the glottic spasm of adults. The dyspnœa is usually neither so acute nor so transient as that of laryngismus stridulus. A smart emetic is the most useful means of allaying the spasm. Mustard or sulphate of zinc may be employed, but ipecacuanha is far the best emetic for the purpose, as its expectorant action is most valuable in the treatment of the accompanying laryngitis. To a child of two years of age it may be given in doses of 5 grains of the powdered root, or drachm doses of the wine, every half-hour until vomiting occurs. The following is recommended by Whitla as acting better than either ipecacuanha or tartar emetic prescribed singly, the dose being for a child one year old:—

℞ Vini antimonialis ... ... ... ♏x.

Vini ipecacuanhæ ... ... ... ♏x.

Syrupi scillæ ... ... ... ... ♏x.

Aquam ad ... ... ... ... ʒj.

One teaspoonful every 15 minutes until vomiting occurs.

In severe cases the inhalation of nitrate of amyl may be tried. Small doses of nitro-glycerine, gr $\frac{1}{500}$, may be given every three hours, and sometimes belladonna proves of value. The remainder of the treatment is that of the laryngitis, which, it should be remembered, is the accompaniment and cause of the spasm. The child must be kept in bed in a warm, well-ventilated room and fed on a light diet; the exhibition of ipecacuanha, or of the mixture given above, should be continued every three hours after the spasm has passed off. The dyspnœa is likely to recur on the next few nights, but tends to get better, and seldom lasts for more than a few days. If the symptoms last longer than this a nourishing diet and stimulants are indicated. In all cases of laryngeal spasm in children, both laryngitis and laryngismus, the tendency to spasm is evoked by some reflex irritation, and the source of this irritation is of the greatest importance in treatment. This is often situated in the alimentary canal, when treatment with rhubarb and soda, or magnesia, combined with the restriction of milk and the avoidance of easily fermentable articles of diet, will have a very good effect. But in a very large number of cases the seat of irritation is in the naso-pharynx; in fact, children subject to laryngeal spasm nearly always suffer from adenoids. Under these circumstances a nasal lotion should be prescribed, to be dropped into the nostrils in infants, and used with a syringe in older children; and after recovery from the attack the adenoids should be removed.

Of the rare form of spasm, dysphonia spastica, the treatment is essentially unsatisfactory, and consists in rest of the voice, improvement in the general health, and especially careful attention to and proper training in production of the voice. There is also no very effective treatment for ictus laryngis; the indications are to avoid any source of irritation, and a course of bromide medication may be tried.

